# The Association of Teen Pregnancy and Violence: A Multilevel Study in Colombia

**DOI:** 10.1089/whr.2021.0075

**Published:** 2024-01-30

**Authors:** Angela Maria Ruiz-Sternberg, Maria Botero-Pinzon, María José Niño-Orrego, Angela Maria Pinzon-Rondon

**Affiliations:** ^1^ Clinical Research Group, Universidad del Rosario, Bogota, Colombia.; ^2^ Department of Biological Sciences, Columbia University College, New York, New York, USA.

**Keywords:** pregnancy in adolescence, exposure to violence, Colombia

## Abstract

**Background:**

Colombia has a high teen pregnancy (TP) rate. In 2018, one in five pregnancies was from teen mothers between 10 and 19 years of age. While TP rates are declining globally, Colombia's TP rate decline has been particularly low, despite sexual education and contraception campaigns. Other factors must be studied to prevent TP. Colombia has a long history of violence. We aim to assess whether there is a relationship between TP and exposure to violence in Colombia.

**Methods:**

Data from the Colombian Demographic and Health Survey 2015 and the Colombian National Department of Statistics were analyzed for association between TP and sexual violence, physical violence, physical punishment as a child, and community violence. Univariate, bivariate, multivariate, and multilevel binary logistic regression models were calculated using SPSS v.25 and HLM v.7.

**Results:**

Fifteen percent of teens were pregnant. Emotional violence was reported by 47%, sexual harassment by 27%, physical violence by 17%, physical punishment as a child by 7%, and unwanted sex by 2%. Unwanted sex (odds ratio [OR]: 3.18, 95% confidence interval [95% CI]: 1.96–5.16), sexual harassment (OR: 2.43, 95% CI: 1.89–3.14), and physical punishment (OR: 20.30, 95% CI: 7.96–22.81) were associated with adolescent pregnancy. In unadjusted models, emotional violence was associated (OR: 1.22, 95% CI 1.06–1.40) and community violence showed a tendency (OR: 1.24, 95% CI: 0.99–1.55). Physical violence was not associated.

**Conclusions:**

Violence exposure and particularly physical punishment, unwanted sex and sexual harassment were associated with TP incidence and should be considered risk factors for TP.

## Background

Adolescent pregnancy is a global health problem that is particularly prevalent in developing countries. In 2016, ∼21 million girls from developing countries, between 15 and 19 years of age (8% of this population) got pregnant, and 3.9 million of them conducted unsafe abortions.^1^ Latin America has the second highest teen pregnancy (TP) rate in the world,^2^ and Colombia is among the countries with the highest rates in the region.^3^ The country is of interest because while teen fertility and TP rates are declining globally,^4,5^ Colombia displays a very mild decrease: from 20.5% in 2005 to 19.5% in 2010 and 17.4% in 2015.^6^ Moreover, throughout 2018, one in five pregnancies were in girls between 10 and 19 years of age,^7^ and almost half of these were undesired, two-thirds were unplanned (64%), and around 70% were conceived with an adult partner.^8,9^

TP has many known negative impacts on socioeconomic development, physical and mental health. According to the World Health Organization, the main cause of death worldwide among girls between 15 and 19 years of age is due to pregnancy and birth-related complications.^10^ Neonates have increased rates of low-birth weight, preterm delivery, and stillborn, among many other problems.^11–13^ Emotional health consequences in teen mothers include depression, stress, anxiety, increased rates of substance abuse,^12,14,15^ and lower scores in the Mental Component Summary—a scale that measures a person's mental health.^16^ In addition, teen mothers are more likely to face stigma or rejection compared to their childless peers, which contributes to emotional health problems.^10,12^

Furthermore, it is common for adolescent girls to drop out of school when they become pregnant, and in general, it is less likely for them to achieve higher education.^10,17^ Lack of education decreases the adolescent mothers' chances of employment, lowers their expected income, and perpetuates the cycle of poverty^10,18,19^; and ultimately, they become a burden for social welfare systems and a loss in tax revenues.^20^

According to the Bronfenbrenner theory, contextual factors can affect a person's health status and wellbeing^21^; one of such contextual factors is violence. Victims of violence are prone to experiencing elevated levels of stress, mental health problems, chronic illness, and other physical conditions.^22^ Some authors have already suggested a possible association between adolescent pregnancy and violence, but the relationship has not been studied in depth.^6,23^ Some types of violence are particularly influential for TP, such as sexual violence, intimate partner violence, child abuse, and community violence. In Nepal, women victims of sexual violence were 2.3 times more likely to report unintended pregnancy.^24^ Yet, there are other types of violence, for which the relationship between TP and violence is debatable. For example, a recent metanalysis study showed a relationship between sexual and physical violence and adolescent pregnancy, while emotional violence and neglect were not related to adolescent pregnancy.^25,26^

Colombia has Latin America's longest history of armed conflict with more than 60 years. It begins by mid-20th century with a clash between Colombia's Conservative and Liberal parties, and the creation of communist guerilla groups in 1958, including the Armed Revolutionary Forces of Colombia, also known as FARC.^27^ Paramilitary groups were created in the 1980s by landowners and drug lords to fight the guerrilla groups, generating even more violence. Despite the recent peace process with FARC in 2016, pervasive violence remains present in the country. Armed conflict persists among the armed forces and the police, and residual guerilla groups, criminal gangs, and drug cartels. Socioeconomic inequality is also extremely high in Colombia, which fuels a troubling situation of social unrest.

Considering the high TP rates in Colombia despite the numerous government campaigns attempted to address them,^6^ and the armed conflict that Colombia has suffered during the last decades, this study aims to determine if violence is a risk factor for adolescent pregnancy in Colombia, and how much different types of violence contribute. The purpose of the study is to determine if there is an association between adolescent pregnancy and exposure to unwanted sex, sexual harassment, physical violence, emotional violence, and physical punishment as a child at the individual level, and violence at the community level.

## Methods

### Study design

It is a quantitative multilevel, national, and cross-sectional study. The study is multilevel because it includes two hierarchic levels, given that individuals are nested in municipalities. The level 1 includes individual information of 13,313 women between 13 and 19 years, who responded the National Demographic and Health survey (DHS) in 2015. The level 2 includes community data of 258 municipalities in Colombia where these women lived from the Colombian National Department of Statistics (DANE, for its acronym in Spanish). A municipality is an administrative territory division with a local government. The study is national because it includes a sample of the Colombian population. Finally, the study is cross-sectional because it was conducted at one point in time.

The protocol was approved by the Universidad del Rosario Institutional Review Board before conducting the research.

### Study area and data sources

Colombia is located in the northern corner of South America. It is divided into 32 departments and 1,103 municipalities. This study uses secondary data from the 2015 DHS and DANE.

The individual-level information comes from the Colombian DHS 2015. This survey was applied to a national representative sample of women between 13 and 49 years of age. It gives information on sexual and reproductive health, maternal and child health, and other important public health measures. The instruments used were based on surveys applied in previous years, adjusted with help of Colombian subject-matter, epidemiology, and biostatistics experts. Researchers piloted the questionnaires in urban and rural areas. The sample came from a master sample created by the Colombian Ministry of Health and Social Protection. It is probabilistic, by clusters (municipalities and household groups), stratified (to represent rural areas and population with unsatisfied basic needs) and multistage.

The universe is the noninstitutionalized civil population from 13 to 49 years of age from 1,122 urban and rural areas, 1,103 municipalities, 18 nonmunicipal areas, and the capital districts of all Colombian departments. Sample framework was obtained from the 2005 Population and Housing Census. Fifty-two thousand four hundred seventy-nine women participated in the Colombian DHS 2015 survey.^8^

The vital records of the DANE contain information on births, fetal and nonfetal deaths, demographics (gender, age) and socioeconomic characteristics, type of event (*e.g.*, cause of death), and place of occurrence, including data at a municipality level. In addition, it serves for change detection in the levels and patterns of mortality and fertility, providing a dynamic view of the population.^7^ All municipality level information comes from this data source.

### Population and sample

The population corresponds to all women between 13 and 19 years of age included in the Colombian DHS 2015, a total of 13,313 women.

We excluded women with no information on their current or previous pregnancy status as well as women with incomplete information on their previous violence exposure, for a final sample of 9,343 adolescent women. Excluded women had similar sociodemographic characteristics as those included in the study.

### Measures

The dependent variable for this study was adolescent pregnancy, whether the woman (13–19 years) is or had been pregnant. It is a dichotomous variable, 1 indicating pregnancy and 0 indicating the contrary.

The individual-level (level 1) variables were unwanted sex, sexual harassment, physical violence, emotional violence, and physical punishment as a child. Unwanted sex shows whether the respondent had ever been forced to have unwanted sex. Sexual harassment presents whether a respondent has been sexually harassed, has received unwelcome sexual comments in the street, or has felt uneasy as a result of a sex-related comment. Physical violence exposes whether a respondent has ever been physically abused –kicked, punched, pushed, slapped, hit with an object, or threatened with a weapon– by a relative or partner.

Emotional violence displays if the respondent reports control issues –over money, family, friends, household decisions, contraceptive use– and/or emotional insults, diminishing messages, bad words, humiliating comments from a relative or partner. Finally, physical punishment as a child describes whether the respondent's parents used to punish her at least once a month by (a) spanking, (b) pushing, or (c) hitting her with an object.

The municipality level (level 2) represents community violence. It refers to the municipality number of homicides per 100,000 inhabitants. The variable tells whether the respondent lives in a municipality with a homicide rate in the upper, medium, or lower third of homicides in the country. The control variables in this section included demographic and socioeconomic factors, along with family, individual, and municipality characteristics.

Demographic and socioeconomic factors include age, ethnicity, information on whether the teen is currently attending school or working, level of education, place of residence, history of migration in the past 5 years, and health provider affiliation. In Colombia, health care affiliation is obtained through three regimes: the contributory regime, which includes employees and people who have the socioeconomic means to pay for health access; the subsidized regime, which is funded by the government for people with no socioeconomic means to pay for health; and the special regime, for people from the military, the police, or the public education system.

Family characteristics are the following: whether the household head is a family relative of the teen, family size, household socioeconomic wealth, if the respondent lived with her biological parents between 12 and 14 years of age, if the teen's mother has four or more children, if the teen's mother has children that exclusively come from the same father, if the teen's mother also had a TP, and if the teen's parents know their teen friends and location.

Finally, individual characteristics include androcentric beliefs, if the respondent has received sexual education information from social media or from close relatives, current knowledge on sexuality, age of menarche, health insurance affiliation, and if the teen has experienced social pressure to initiate sexual life.

Municipality characteristics—unsatisfied basic needs.

### Data analysis

For data processing, we used the statistical software SPSS v.25 [Statistical Power for the Social Sciences]. We cleaned and merged databases, selected the data required for the analysis, verified its accuracy applying filters and recoded the selected variables for analysis.

Initially, we conducted a descriptive analysis to characterize the study population using frequencies and proportions for categorical variables. Using minimum value, maximum value, and measures of central tendency for numerical variables, this analysis was conducted in the whole population accounting for pregnancy status. The difference between pregnant and not pregnant (NP) adolescents was calculated with bivariate analysis; contingency tables and chi-squares for categorical variables, and independent-sample *t*-tests or Mann–Whitney tests for numerical variables. We ran a multivariate analysis using binomial logistic regression to adjust a first-level model.

We fitted multilevel logistic regression models using the software HLM SSI v.7 [Hierarchical Linear and non-Linear Models—Scientific Software International] to explore the association between violence at a municipality level, represented as the rate of homicide, and adolescent pregnancy. Odds ratio (OR) and confidence intervals (CI) were calculated. We conducted interaction analyses of the different types of violence with demographic and socioeconomic factors and individual characteristics. Finally, we ran two sensitivity analyses with a randomly selected sample of the entire population to rule out the effects of oversampling bias using bootstrapping with the statistical software R.

## Results

### Description of the sample

Pregnancy was reported by 15% of teens. The average age of the studied population was 15.9 years, 17.7 for pregnant, and 15.6 for NP women. Most of the population belonged to the ethnic majority (76%), lived in urban areas (73%), and was affiliated to the subsidiary health system (65%). See Table 1 for a complete description of the sample. We found important differences between the pregnant and NP teens, while 86% of NP teens were attending school, only 29% of pregnant teens were doing so. On educational attainment, 9% of NP teens reported reaching only elementary education and 16% of the pregnant teens reported the same.

**Table 1.** Descriptive Statistics of Sociodemographic and Sexual and Reproductive Characteristics of Colombian Teens

Variable	Frequency	Proportion	Mean	Min	Max
Pregnancy	1,443	0.15			
Age			15.9	13	19
Ethnicity					
Majority	7,128	0.76			
Indigenous	1,157	0.12			
Black	923	0.10			
Other	135	0.01			
Currently attending school	7,201	0.77			
Education					
Elementary	981	0.10			
High school	7,459	0.80			
Higher	903	0.10			
Respondent currently working	1,630	0.17			
Type of place of residence (1 = Urban)	6,863	0.73			
Has migrated in the past 5 years	1,805	0.19			
Household head is a relative	8,200	0.88			
Family size					
Small		0.22			
Medium		0.58			
Large		0.21			
Household wealth					
Poorest	2,670	0.29			
Poor	2,905	0.31			
Middle	1,766	0.19			
Richer	1,222	0.13			
Richest	780	0.08			
Lived with biological parents from 12 to 14 years	3,503	0.37			
Mother has 4 or more children	4,351	0.47			
Mother's children from same father	5,510	0.59			
Mother had a teen pregnancy	4,747	0.51			
Menarche					
Before 12 years of age	2,209	0.24			
Between 12 and 14 years of age	6,407	0.69			
After 15 years of age	727	0.08			
Health system affiliation					
Contributory regime	2,586	0.28			
Subsidized regime	6,037	0.65			
Special regime	245	0.03			
No affiliated	475	0.05			
Parents know teen's friends and location (*N* = 8,409)			2.55	0.0	3.0
Androcentric beliefs			0.54	0.0	1.8
Received information on sexuality form media			1.48	0.0	3.0
Traditional opinions on sexuality (*N* = 8,975)			5.39	0.0	18.0
knowledge on sexuality			0.50	0.0	1.0
Social pressure to have sexual relations			0.64	0.0	3.0

Pregnant teens were more likely to be working (25%) in comparison to NP teens (16%). While 94% of NP teens were living with a blood relative, 53% of the pregnant ones were doing so. We also found differences on wealth and health system affiliation, with a higher rate of pregnant women found in the lower quintiles of wealth, 74% pregnant versus 57% NP, and a lower rate of pregnant women affiliated to the contributory regimen, and 17% pregnant versus 30% NP.

At the municipality level, the percentage of people with unsatisfied basic needs spanned from 5% to 100%, with a mean of 42% and a median of 39%.

### Adolescent pregnancy and violence

The adolescent women included in the study reported emotional violence (47%), sexual harassment (27%), physical violence (17%), physical punishment (7%),s and sexual violence (2%) (Table 2).

**Table 2.** Descriptive Statistics of Violence Exposure in Colombian Teens

Variable	Frequency	Proportion
Sexual violence	190	0.02
Sexual harassment (*N* = 9,296)	2,507	0.27
Physical violence (*N* = 9,246)	1,544	0.17
Emotional violence	4,409	0.47
Physical punishment	664	0.07

Chi-square tests document significant associations (*p* < 0.001) between TP and sexual violence, sexual harassment, emotional violence, and physical punishment. We did not, however, find association between physical violence and adolescent pregnancy.

### Regression analysis assessing associations between adolescent pregnancy and violence

The results of the binomial logistic regression of the individual—level 1 variables, on adolescent pregnancy, are shown in Table 3. The initial model included only the violence variables, and the adjusted model included all the studied variables. Physical punishment was strongly associated with adolescent pregnancy, although the magnitude of association decreased after controlling for all the studied variables, it continued showing a very strong positive association (OR: 7.61, 95% CI: 5.32–10.90). Sexual violence also showed a positive association that remained after adjusting the model (OR: 1.95, 95% CI: 1.18–3.22). Moreover, sexual harassment and emotional violence showed an association in the unadjusted model (only violence variables), yet, this association was lost after adjusting the model. Finally, physical violence did not show any association to adolescent pregnancy.

**Table 3.** Unadjusted and Adjusted Binomial Logistic Regression Level 1 Model of Teen Pregnancy on Exposure to Different Types of Violence

	Raw model				Adjusted model			
	OR 95% CI				OR 95% CI			
	OR	Inferior	Superior	Sig.	OR	Inferior	Superior	Sig.
Sexual violence	2.73	1.91	3.89	<0.001	1.95	1.18	3.22	<0.01
Sexual harassment	2.55	2.22	2.92	<0.001	1.29	1.05	2.47	0.179
Physical violence	0.90	0.75	1.07	0.239	1.02	0.80	1.32	0.857
Emotional violence	1.22	1.06	1.40	0.006	0.92	0.75	1.14	0.457
Physical punishment	19.97	16.27	24.51	<0.001	7.61	5.32	10.90	<0.001
Age					1.69	1.57	1.82	<0.001
Ethnicity majority					Reference category			
Ethnicity indigenous					0.87	0.63	1.21	0.419
Ethnicity Black					1.11	0.82	1.50	0.503
Ethnicity other					1.07	0.50	2.31	0.853
Currently attending school					0.29	0.23	0.35	<0.001
Elementary education					3.73	2.29	6.08	<0.001
Highschool education					2.37	1.73	3.25	<0.001
Higher education					Reference category			
Respondent currently working					0.74	0.58	0.93	0.009
Type of place of residence					0.92	0.68	1.25	0.599
Has migrated in the past 5 years					0.77	0.60	0.99	0.044
Household Head is a Relative					0.37	0.27	0.52	<0.001
Small family size					0.36	0.26	0.50	<0.001
Medium family size					0.60	0.48	0.75	<0.001
Large family size					Reference category			
Poorest					1.69	0.98	2.89	0.05
Poor					1.70	1.07	2.69	0.024
Middle					1.34	0.83	2.16	0.226
Richer					1.62	1.00	2.62	0.05
Richest					Reference category			
Lived with biological parents from 12 to 14 years of age					0.92	0.75	1.14	0.470
Mother has 4 or more children					1.01	0.81	1.25	0.953
Mother's children from same father					0.88	0.71	1.09	0.251
Mother had a teen pregnancy					1.42	1.17	1.73	<0.001
Parents know teen's friends and location					0.76	0.66	0.88	<0.001
Androcentric beliefs					2.21	1.48	3.29	<0.001
Received information on sexuality from media					1.05	0.96	1.16	0.266
Traditional opinions on sexuality					0.95	0.91	0.98	<0.001
knowledge on sexuality					0.77	0.47	1.27	0.300
Menarche before 12 years					3.95	2.47	6.34	<0.001
Menarche between 12 and 14 years					2.44	1.57	3.79	<0.001
Menarche after 15 years					Reference category			
Affiliated to contributory regimen					Reference category			
Affiliated to contributory regimen					1.24	0.96	1.61	0.098
Affiliated to contributory regimen					1.27	0.63	2.54	0.507
No affiliated					0.53	0.32	088	0.013
Social pressure to have sexual relations					2.31	1.91	2.79	<0.001

95% CI, 95% confidence interval; OR, odds ratio.

### Multilevel regression models

Table 4 presents the results of the multilevel analysis. In the unadjusted model, we found associations between sexual violence (OR: 2.69, 95% CI: 1.79–4.03), sexual harassment (OR: 2.55, 95% CI: 2.16–3.02), emotional violence (OR: 1.24, 95% CI: 1.07–1.44), and physical punishment (OR: 10.30, 95% CI: 6.38–15.16) with TP. High levels of violence at the municipality level presented a tendency associated with TP; however, it was not statistically significant (OR: 1.24, 95% CI: 0.99–1.55).

**Table 4.** Unadjusted and Adjusted Bernoulli Multilevel Regression Models of Teen Pregnancy on Exposure to Different Types of Violence

	Raw model				Adjusted model			
	OR 95% CI				OR 95% CI			
	OR	Inferior	Superior	Sig.	OR	Inferior	Superior	Sig.
Level 1								
Sexual violence	2.69	1.79	4.03	<0.001	3.18	1.96	5.16	<0.001
Sexual harassment	2.55	2.16	3.02	<0.001	2.43	1.89	3.14	<0.001
Physical violence	0.90	0.71	1.14	0.380	1.09	0.80	1.49	0.604
Emotional violence	1.24	1.07	1.44	0.006	1.25	0.99	1.69	0.084
Physical punishment	10.30	6.38	15.16	<0.001	14.38	7.96	22.81	<0.001
Level 2								
Low municipality violence rate	Reference category				Reference category			
Medium municipality violence rate	1.06	0.86	1.32	0.578	1.07	0.69	1.26	0.563
High municipality violence rate	1.24	0.99	1.55	0.057	1.22	0.62	1.10	0.280
Reliability: 23%								

In the adjusted models, the associations of sexual violence (OR: 3.18, 95% CI: 1.96–5.16), sexual harassment (OR: 2.43, 95% CI: 1.89–3.14), and physical punishment (OR: 14.38, 95% CI: 7.96–22.81) with TP remained. However, the tendency between violence at the municipality level and TP was lost.

### Interaction and sensitivity analysis

We conducted multiple interaction analysis of sexual violence, sexual harassment, emotional violence, and physical punishment with age, education, ethnicity, health system affiliation, and household wealth, and we did not find any interaction effect. We also conducted 2 sensitivity analysis using bootstrapping techniques with randomly selected subsamples containing 500 adolescent girls of the total population, one of the analysis was focused on the logistic regression and the other on the multilevel modeling, finding essentially the same results that are presented above.

## Discussion

This study examined the hypothesis that adolescent women exposed to violence are more likely to get pregnant in Colombia. Although we cannot draw a causal conclusion from it, the study presents an association between exposure to sexual violence, sexual harassment, and physical punishment during childhood and TP. Unlike many studies on this topic, this analysis incorporates community violence as a contextual risk factor.

### Sexual violence: sexual harassment and adolescent pregnancy

Sexual violence is a worldwide phenomenon that affects one in every five children or adolescent girls.^28^ This study found an association between sexual violence and adolescent pregnancy (OR: 3.18, 95% CI: 1.96–5.16), with slightly higher rates than previously reported by other authors. A meta-analysis by Noll et al., found that sexual abuse increased the probability of TP by 2.21-fold and a meta-analysis by Madigan et al. found a 2.06-fold increase.^26,29^ Sexual violence, the use of force or coercion to obtain a sexual act, could be directly or indirectly related to adolescent pregnancy. Directly, because when sexual intercourse is forced, women tend to be unprotected against a pregnancy, since this is usually an unexpected event.^23^ Indirectly, because the trauma of sexual abuse is so great that it changes the behavior of the victims.

There is enough evidence that shows an association between sexual abuse, substance abuse, and sexual risk behaviors.^30^ Furthermore, women who suffer sexual violence find it hard to denounce out of fear. Perpetrators might dismiss the allegation, hurt them if they do, or make them feel inferior.

Sexual violence is a problem in Colombia that has been reported mainly in girls between 10 and 16 years of age; it has been linked to the unintended pregnancy of young women.^23,31^ Rape has been used as a weapon of war in the Colombian armed conflict.^24^ A study based on the Colombian Demographic and Health Survey of 2005 found that Colombian female youth, between the ages of 13 and 24, who had been victims of sexual violence, were significantly more likely to report an unintended pregnancy compared to those who had not been victims of sexual violence.^24^

Sexual harassment was also associated with TP in this study (OR: 2.43, 95% CI: 1.89–3.14). Although sexual harassment is recognized today as a form of abuse and it is at the forefront of discussions, still it is hard for its victims to come forward. Some people argue that sexual harassment claims are exaggerated or misinterpreted by the victims, yet, those affected suffer a great trauma.^32,33^

### Physical punishment as a child and adolescent pregnancy

Physical punishment as a child can be considered a form of child abuse.^34^ This variable was found to have the biggest association with TP in our study (OR: 14.38, 95% CI: 7.96–22.81). Child abuse creates a negative household environment that leads to depression and dissociation, and consequently can cause adolescents to try to escape: by getting a romantic partner or spouse and leaving the household, joining a gang, committing suicide, and/or falling into substance abuse, among others.^31^

The most frequent perpetrator of the abuse is the victim's parent. Adams found that the frequency of any past abuse (especially physical abuse) is significantly higher in adolescents who had ever been pregnant compared to the never pregnant group (58% vs. 38%, respectively).^35^

Physical abuse is a contextual factor that becomes especially relevant in vulnerable populations that reside in politically unstable developing countries; a study of 26,055 adolescent girls found a high prevalence of TP in women exposed to physical violence compared to those who were not, with approximately a five-fold increase.^36^

Garwood et al., found that child maltreatment was associated with TP independently of poverty (OR: 1.66).^25^ Hillis et al. found that negative household environments have a cumulative risk effect on TP.^37^

The use of physical punishment as a child rearing practice has been common in Colombia. When this physical punishment becomes frequent, children seek out mechanisms to leave their households and pregnancy is one of them. The introduction of Children's Rights instruction in elementary school curriculums has empowered children to look for nonabusive environments.

### Community violence and adolescent pregnancy

Violence related to negative community environments includes direct victimization, witnessing, and hearing about violent acts in the community, such as criminality, interpersonal aggressiveness, delinquency, and conduct problems.^38^ People living in toxic environments tend to have short life expectancies. This leads them to develop a feeling of urgency to accomplish their life goals as fast as possible, that is, having children.^39,40^ In addition, many violent communities lack social services because it is hard to implement and maintain them safely in the community, such as lack of sexual education programs, because it is hard for these to get into places dominated by gangs, which increases the chances of adolescent pregnancy. It is also common that in many of these violent communities, it is hard to find quality schools and other educational institutions.^38,41^

A study conducted in the United States reported community violence exposure as a public health problem. Over 85% of urban youth report witnessing some form of community violence in their lifetime and almost 70% report direct victimization. Adolescents have a higher risk of being negatively affected by community violence. Poor health outcomes include post-traumatic stress, depression, and anxiety.^38^ In addition, a lower rate of positive youth development is reported, encompassing not only mental health, but school development, caring for others, sense of positive self-worth, and self- efficacy.

Colombia is a particularly violent country. Since 1830, over 10 civil wars took place; violence caused ∼400,000 reported deaths, and even more missing. Violence has become a part of the culture.^42^ Our study shows a tendency toward TP risk in our unadjusted models; however, it was not statistically significant and the tendency was lost in adjusted models.

### Limitations

Although we have a very good sample size that is representative of the Colombian population, this study has multiple limitations. The cross-sectional data did not permit us to explore causality nor the specific mechanism that explains the association. It is not possible to establish the temporal order of pregnancy and violence exposure. In addition, the municipality information gathered came from young women who resided in the location at the time of the survey, yet, this may not be the same municipality where she got pregnant. Finally, given both TP and violence are sensitive topics and the study is based on self-report, it is likely that they were underreported.

### Recommendations

Violence exposure should be actively searched by health care professionals and as public health policies to reduce the rate of TP, and its associated short- and long-term complications.

We recommend a cohort study to determine association between TP and sexual violence—harassment and physical punishment as a child. This study suggests that violence prevention strategies could be important to prevent TP.

## Conclusion

Findings from this representative sample of adolescent women in Colombia support the hypothesis that sexual violence, sexual harassment, and physical punishment as a child could be significant risk factors for TP. TP causes health problems on the teen mother and the infant, as well as a socioeconomic burden on the system. Colombia, a country internationally recognized for its long-standing civil war and violence requires constant screening for violence, especially sexual violence, sexual harassment, and physical punishment as a child. Any teen exposed to violence should be supported, and proper assistance should include strategies to prevent TP.

## Abbreviations Used

95% CI: 95% confidence intervals
DHS: Demographic and Health survey
NP: not pregnant
OR: odds ratio
TP: teen pregnancy
